# Da Vinci's mischief: xylem conduits in the stems of woody plants do not furcate

**DOI:** 10.1111/nph.71097

**Published:** 2026-03-24

**Authors:** Gilberto Alemán‐Sancheschúlz, Tommaso Anfodillo, Ana Isabel Pérez‐Maussán, María Magdalena Ayala‐Hernández, Mark E. Olson

**Affiliations:** ^1^ Instituto de Biología, Universidad Nacional Autónoma de México, Tercer Circuito s/n de Ciudad Universitaria CDMX 04510 Mexico; ^2^ Unidad de Investigación en Sistemática Vegetal y Suelo, Facultad de Estudios Superiores Zaragoza, Universidad Nacional Autónoma de México, Batalla 5 de Mayo s/n, Ejército de Oriente CDMX 09230 Mexico; ^3^ Department Territorio e Sistemi AgroForestali, University of Padova Legnaro Padua 35020 Italy

**Keywords:** carbon economy, conduit widening, da Vinci's rule, furcation, hydraulic architecture, natural selection, Pipe Model, xylem

## Abstract

- The hydraulic architecture of plants is often modeled as a ‘furcating’ network, in which xylem conduits proliferate in number toward the stem apex, analogous to animal circulatory systems. Yet whether furcation actually occurs within woody stems remains untested, despite major implications for carbon costs and hydraulic efficiency.
- We measured the number and diameter of functional xylem conduits at the base and tip of unbranched stem segments in 112 woody species spanning 57 families and 31 orders. Standard and phylogenetically informed regressions were used to evaluate relationships between conduit number, stem length, and tip‐to‐base conduit widening.
- Conduit number remained constant between base and tip, showing no evidence of furcation, whereas conduit diameter increased predictably with distance from the apex, with a slope of 0.22, closely matching theoretical expectations for hydraulically optimal widening.
- These results support a ‘Widened Pipe Model’ of xylem architecture, in which conductance is maintained through conduit widening rather than multiplication. We suggest that the absence of furcation suggests that da Vinci's rule of area‐preserving branching does not apply within stems, emphasizing the need to revisit core assumptions in models of tree hydraulic design.

## Introduction

An important participant in the carbon economy of trees is the number and size of xylem conduits. Conduits widen from the apex to the base of the stem, which may help maintain sapwood carbon costs constant per unit leaf area as trees grow taller (Anfodillo & Olson, 2024; Berry *et al*., 2024). Understanding how conduit widening participates in tree carbon budgets is therefore important for identifying the factors that determine whether sink carbon costs per unit leaf area remain constant or increase with height, and ultimately for uncovering the factors of natural selection that shape plant form and function.

**Fig. 1:**
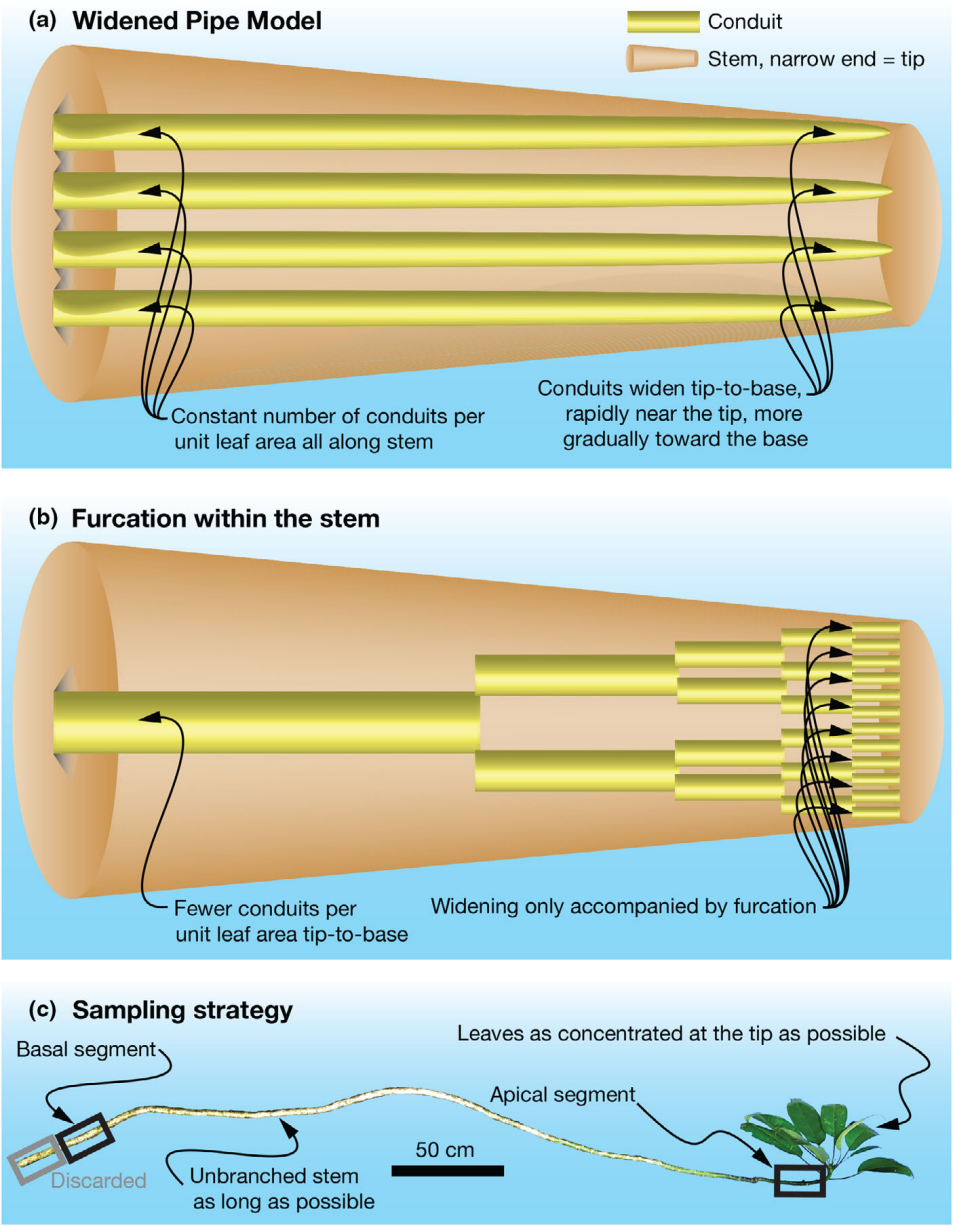
Widened Pipe Model vs furcation in the stem, and sampling strategy to distinguish between them. (a) The Widened Pipe Model predicts a constant number of conduits per unit leaf area along the stem. The hydraulic resistance that accumulates with increasing path length during height growth is offset by conduit widening from tip to base, rapid near the stem tip and progressively slower toward the base. (b) In furcation scenarios such as those predicted by Murray's law, conduit diameter changes only where conduits divide. Hydraulic resistance is reduced by the distribution of flow into a greater number of conduits distally, while carbon costs are minimized by the coalescence of conduits basipetally. (c) To distinguish between these possibilities, we sampled long, unbranched terminal shoots to determine whether conduit number varies along stems for the same leaf area. We selected shoots with leaves as concentrated as possible at the tip, since any furcation present should be inversely proportional to tip‐to‐base widening. Because widening is most rapid near the tip, shoots with leaves distributed all along the stem would reduce the likelihood of detecting furcation by shifting sampling below the zone where widening, and any associated furcation, occurs. Long unbranched stems with tightly clustered leaves therefore maximize the likelihood of detecting any furcation. The basal (black rectangle) and apical (black rectangle at right) segments were used for staining and quantification of functional conduits; the basal segment (gray rectangle) was discarded. This design directly tests whether the number of active vessels increases toward the tip, the core prediction of the furcation hypothesis.

One aspect of conduit widening potentially contributing to stem carbon cost is whether conduits ‘furcate’ in the stem as they do in leaves, producing either a ‘top‐heavy’ system (higher carbon cost) or not (lower cost, see Anfodillo & Olson, 2024). Conduits definitely widen tip‐to‐base (Fig. 1a), but it remains unclear whether this widening is also associated with conduit furcation (Fig. 1b), an increase in the number of conduits per unit leaf area from base to apex (Rosell & Olson, 2019). Furcated conductive systems are ones in which any change in conduit diameter involves change in conduit number between successive levels of branching of the conductive system. Xylem conduit furcation reported so far has been observed mostly in the distal parts of leaves, where water leaves the xylem from the highly branched conduits analogous to animal capillaries (McCulloh *et al*., 2009; Gleason *et al*., 2018; Lechthaler *et al*., 2019; Rosell & Olson, 2019; Anfodillo & Olson, 2024).

It is possible that conduits in stems also furcate, maintaining conductive capacity as flow divides distally, as in leaves and animal vasculature (McKown *et al*., 2010; Price *et al*., 2013; Jyske & Hölttä, 2015; Rosenberg, 2022). If such a hierarchy operates in stems, it could constitute a general scaling principle in plants, allowing water supply to keep pace with increases in path length and demand. However, for the same basal conduit number, furcation imposes greater construction cost than a constant‐number design, because partitioning lumen area into many distally narrower conduits increases the summed conduit perimeter and therefore cellulose and lignin investment. In addition, because frequent pit crossings add series resistance, furcation, with its numerous conduit‐to‐conduit junctions, could, in the absence of sufficient compensatory widening, yield higher hydraulic resistance for the same or greater carbon cost than a nonfurcated architecture. By contrast, conduits that widen tip‐to‐base along the stem, together with any mechanisms of ultra‐widening permeability increase (Anfodillo & Olson, 2024), in principle can achieve comparable hydraulic performance at lower carbon cost, making conduit proliferation unnecessary.

Most recent studies that model tree structure and function engage with the possibility of conduit furcation, suggesting that furcation in the stem has been widely regarded as plausible. These models, inspired by animal vasculature, posit hierarchical branching that balances maintaining high conductance against the carbon cost of constructing conduit volume, consistent with Murray's law scaling and approximately constant conductance across branching levels (McCulloh *et al*., 2003, 2009; McCulloh & Sperry, 2005). In plant studies, recent models of stem structure often draw on theoretical frameworks that include conduit furcation (McCulloh *et al*., 2003, 2004, 2009; McCulloh & Sperry, 2005; Mencuccini *et al*., 2007; Atala & Lusk, 2008; Lintunen & Kalliokoski, 2010; Savage *et al*., 2010; Hölttä *et al*., 2011, 2013; Denny, 2012; Sperry *et al*., 2012; Bentley *et al*., 2013; Smith *et al*., 2014; Jyske & Hölttä, 2015; Petit *et al*., 2023; Sopp & Valbuena, 2023). Furcation has been empirically confirmed in leaves with reticulate or dichotomizing venation, where conduits proliferate toward their narrow termini (McCulloh *et al*., 2003, 2009; Lintunen & Kalliokoski, 2010; Gleason *et al*., 2018; Lechthaler *et al*., 2019).

A few studies question the likelihood of extensive furcation in the stem, suggesting that if it exists, it may be restricted to the distalmost parts of the conductive stream (Petit *et al*., 2008, 2010; Denny, 2012; Koçillari *et al*., 2021; Sopp & Valbuena, 2023). McCulloh & Sperry (2005), for example, note that if there is any furcation at all in the stems of woody plants, it is likely to be much lower than leaves. By contrast, explicit rejections of furcation in stems are rare (Petit *et al*., 2008, 2010), although Mencuccini *et al*. (2007, p. 1090) note that ‘existing data do not generally support the presence of conduit furcation in the stems of trees’.

In this study, we experimentally tested the hypothesis that xylem conduit number increases from the base toward the tip of woody stems. To provide a broad test of furcation, we quantified the number of functional xylem conduits at the base and apex of unbranched stems of 112 plant species, spanning 57 families and 31 orders of vascular plants. This extensive taxonomic and ecological sampling allowed us to assess whether furcation occurs across a wide diversity of growth forms, wood densities, and environments. Our results confirm that the number of functional xylem conduits per unit leaf area remains constant from the base toward the tip of the stems. We discuss our results in the context of our ‘stretched’ hypothesis of plant hydraulic evolution (Anfodillo & Olson, 2024) and propose that the lack of furcation in stems suggests that woody plants deviate from da Vinci's ‘rule’ of conservation of cross‐sectional areas of stems.

## Materials and Methods

### Study sites and studied species

**Fig. 2:**
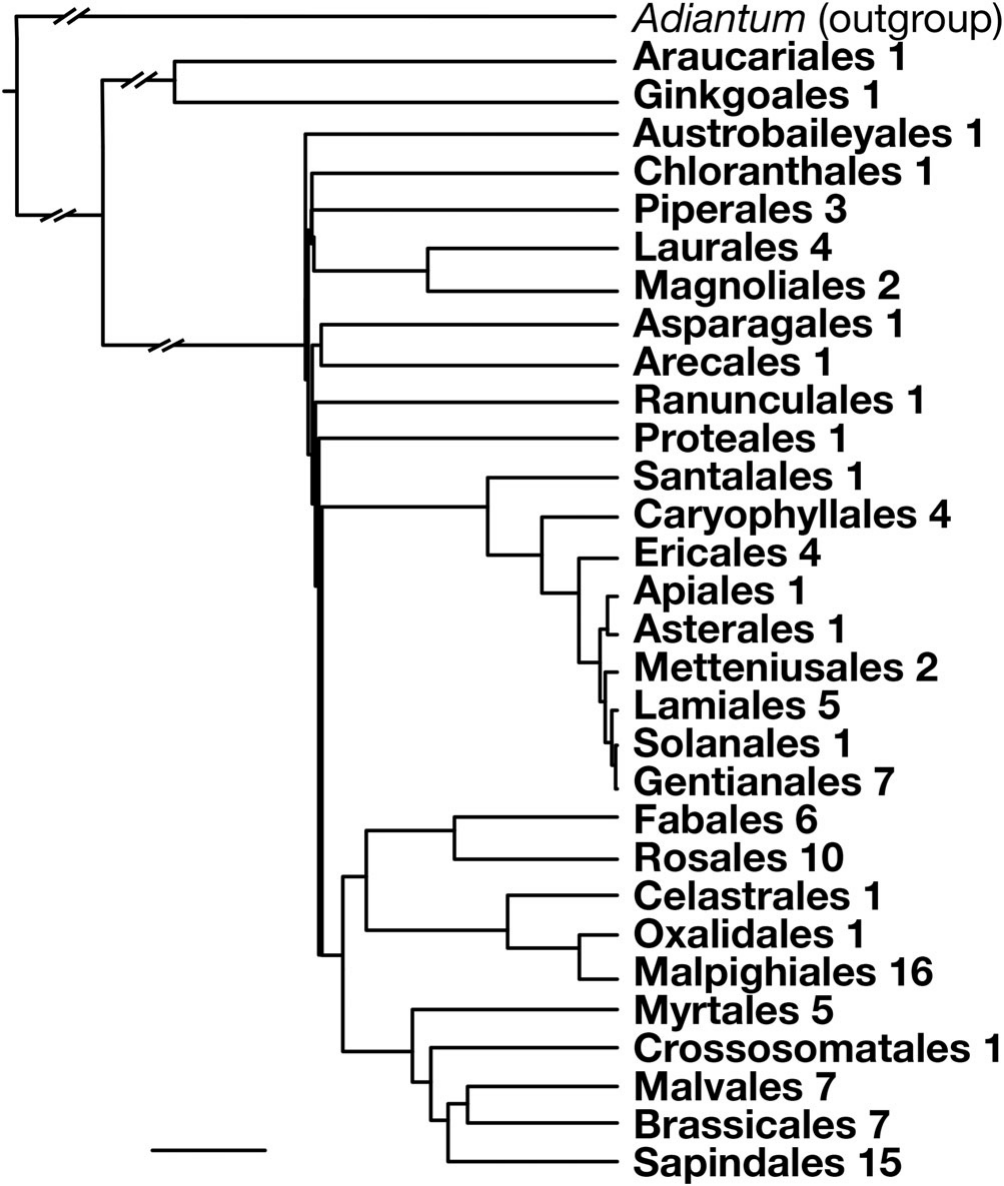
Sampling across a wide phylogenetic coverage. This phylogeny of our sampled species illustrates that our sampling covered 31 orders and 112 woody species in 57 families. This broad phylogenetic coverage ensures that the relationships we identify are not restricted to a single lineage but reflect patterns likely shared across major branches of seed plant evolution. Numbers indicate the number of species in each order. The bar at lower left indicates 50 Myr. Long branches are truncated for clarity.

We selected sites that offered both high morphological and phylogenetic diversity (Fig. 2) and laboratory space for conduit staining and sectioning. Sampling took place at two Universidad Nacional Autónoma de México field stations, the tropical rainforest at Los Tuxtlas (41 species) and the tropical dry forest at Chamela (55 species), as well as at the Francisco Javier Clavijero Botanical Garden of the Instituto de Ecología, Veracruz (16 species). In total, we sampled 112 vascular plant species spanning 57 families and 31 orders (Supporting Information Table S1).

### Functional conduits

Measuring total conduit number in whole plants is impractical because it would require identifying every functional conduit both at the stem base and at each branch tip. By contrast, measurements along individual branches provide a tractable and appropriate test for furcation. Branches are appropriate because conduit widening is greatest toward branch tips (Olson *et al*., 2021), and furcation should be closely correlated with widening (McCulloh & Sperry, 2005; Hölttä *et al*., 2011). An inverse relationship between widening and conduit furcation emerges naturally when cross‐sectional area (as in da Vinci's rule) or volumetric flow (as in Murray's Law) is conserved. In such systems, hydraulic capacity can be maintained either by increasing the number of conduits or by increasing their diameter, such that widening and furcation represent complementary means of sustaining water transport along the path length. To provide situations in which furcation should be most apparent, we selected terminal branches (i.e. ones ending in leaves) spanning from 0.6 to 7.7 m in length, precisely the size range over which widening, and therefore furcation, if present, should be most marked.

Variation in leaf area along a branch can complicate tests for conduit furcation because if leaves or leaf‐bearing branches are inserted all along the stem, then conduit number will naturally vary given the relationship between conduit number and evaporative surface. In such cases, variation in conduit number reflecting greater leaf demand would need to be separated from that due to furcation (Lintunen & Kalliokoski, 2010). To minimize this confounding effect, we selected unbranched shoots that were as long as possible from the tip toward their insertion either to a larger branch or to the root collar, and that carried their leaves in as short a length as possible toward the distal end (Fig. 1c). We then sampled the xylem at the base of the branch, above its insertion point, and at the branch tip immediately below the leaf‐bearing region, ensuring that both measurements represented conduits supplying the same leaf area.

Another potentially confounding factor is that inactive conduit number can also increase toward the base of branches, as older, nonfunctional vessels accumulate over time and contribute to the total conduit count without participating in water transport (Jacobsen *et al*., 2015). Simply counting all the conduits present at tip and base would risk including these inactive conduits in our counts, potentially leading to overestimation of basal conduit number and biasing our results away from detecting any furcation present. To avoid overestimating conduit number at the branch base, we used a method for identifying active conduits by passing an aqueous safranin solution through the xylem conduits. This technique allowed us to visualize, count, and measure only those conduits that were functional along the length of a segment, excluding nonfunctional, embolized, or otherwise occluded conduits from our analysis. Ideally, we would have relied on transpirational pull to draw the staining solution naturally through the entire branch, simulating actual sap flow conditions (Hargrave *et al*., 1994; Jacobsen *et al*., 2007; Jacobsen & Pratt, 2012). However, in practice, this proved unfeasible for many of the species included in our study, because achieving continuous passage of the solution to the leaves was difficult through segments often several meters long. Ensuring broad phylogenetic coverage and capturing a wide diversity of ecological strategies, such as variation in wood density, leaf size, leaf mass per area, and growth habit, also required us to collect branches from relatively remote locations, often relatively distant from the laboratory facilities. Therefore, adopting a controlled staining method in the field was essential for standardizing conduit visualization across the diverse range of species and environments included in our sampling.

Therefore, we chose a method compatible with fieldwork conditions. First, we cut the selected branch and immediately sealed the cut end with liquid silicone and covered it with a small water‐filled balloon, secured with a rubber band, and transported it to the laboratory as quickly as possible, usually within 1 or 2 h. All subsequent cuts were made underwater. Next, we cut and discarded the first 15 cm from the base to avoid including air‐filled conduits in our analyses. Afterward, from each branch, we cut two segments between 10 and 15 cm in length: one just above the discarded section (basal segment) and another just below where the leaves started (apical segment) (Fig. 1c). We attached 10 cm pieces of latex tubing to the distal end of each stem segment, ensuring that the proximal end, corresponding to the root side, was connected in order to maintain the natural direction of water flow from roots to leaves. The tubing was then filled with a filtered 0.1% aqueous solution of safranin O (Sigma‐Aldrich). Connections were checked for leaks and sealed with Parafilm (Bemis Co. Inc., Neenah, WI, USA) when necessary. We avoided using clamps to prevent excessive pressure on the stem segment, which could alter water flow through the xylem conduits from the last growth period, where most water transport to the leaves likely occurs (Ellmore & Ewers, 1986; Bodo & Arain, 2021; Xiang *et al*., 2023). As safranin solution levels in the tubes decreased due to atmospheric pressure, we gradually refilled the tubes until the stain solution appeared at the opposite end of the segment, which was kept submerged in water for the entire duration of the staining process. The close 1 : 1 correspondence between conduit number at the base and tip discussed below indicates that our procedure maintained xylem functionality throughout sampling and staining. Significant methodological artifacts would certainly disturb, not reinforce, such proportionality, supporting the appropriateness of our methodology.

Two sampling artifacts, open vessels and cutting‐induced embolism, would both tend to lower basal conduit counts relative to apical counts and thus mimic furcation. Open‐vessel effects arise when vessels longer than the segment admit air (Sperry, 2013; Lens *et al*., 2022). Because vessel length generally increases from tip to base (Comstock & Sperry, 2000), the basal segments should be more susceptible. Even cutting underwater can seed embolism at the cut surface (Wheeler *et al*., 2013), a risk greater for wider conduits. Because conduits are wider in the basal segments, this possible artifact would also affect the basal segments more than the apical ones. In both cases, if present, these artifacts would reduce the number of functional conduits observed at the base relative to the tip, creating an apparent excess of conduits toward the apex. This artefact would generate a pattern *rejecting* our expectation of equal conduit numbers at tip and base, making our design conservative with respect to our hypothesis. In other words, any bias introduced by open vessels or cut‐induced embolism would work against detecting the predicted 1 : 1 relationship. As described below, our results showed no such discrepancy, indicating that open‐vessel artefacts did not affect our conclusions. Importantly, our tracheid‐bearing species in *Podocarpus matudae* Lundell and *Ginkgo biloba* L. provide strong internal controls: their extremely short tracheids mean that re‐excision of 15 cm eliminates open conduits outright, yet these species show the same 1 : 1 pattern.

After staining the functional conduits, we cut each segment in half using either a hand saw or pruning shears and then trimmed the cut surfaces with a single‐edge razor blade for an initial inspection of the staining results. Next, we obtained transverse sections using a sliding microtome. We flattened and dried the sections by placing them between the pages of hardcover notebooks, then stored the sections in plastic zip bags. In the laboratory, we placed the sections in 20 ml scintillation vials with xylene for 2 d; we then dried the excess xylene with a paper towel and mounted the sections on glass slides using synthetic resin. We examined the sections under a Z45L stereo microscope (Cambridge Instruments, Shanghai, China) and photographed each entire section using a Sony A6000 digital camera (Sony Corp., Tokyo, Japan). From the photographs, we recorded the number of functional (stained) conduits and measured the lumen diameter of 30 of them. We performed these measurements using ImageJ 1.53k (National Institutes of Health, Bethesda, MD, USA), calibrating the images with a Nikon stage micrometer (MBM13100; Nikon Corp., Tokyo, Japan) to convert pixel values to micrometers. All photographs are compiled and available at https://doi.org/10.6084/m9.figshare.30569648, and all quantitative variables that were recorded are included in Dataset S1.

While our primary analyses used unbranched stems to minimize complexity of processing, the same logic and protocol extend to branched stems. For a branched stem *N*_base_ is the number of functional conduits at the base of the parent segment, just as in unbranched stems, whereas *N*_tip_ is the sum of functional conduits summed across all daughter twigs. Furcation refers to a coupled change in conduit number and diameter, in which a reduction in diameter is accompanied by a compensatory increase in conduit number. Because furcation concerns the internal scaling relationship between conduit number and diameter, rather than the presence or absence of external shoot branching, the outer branching structure should not in itself influence whether furcation occurs. If external branching does not itself generate conduit proliferation within the stem, the *N*_tip_ *–N*_base_ relationship should remain one to one as in unbranched paths. As a check, we applied the same staining and counting procedure to three branched stems of *Wigandia urens* (Ruiz & Pav.) Kunth (Boraginaceae), comparing the parent segment with the summed daughters. If external branching does not participate in conduit furcation, the *Wigandia* points should fall with the unbranched stem data.

### Statistical analyses

#### Standard linear models

We conducted three regression analyses to evaluate patterns in the abundance and diameter of functional xylem vessels along the stem. All variables were log_10_‐transformed before analysis (Kerkhoff & Enquist, 2009). This transformation was applied to all anatomical and structural variables, including the number and diameter of xylem functional conduits at the base and tip of the stems, segment length, and the distance from each segment to the apex. This transformation is appropriate because these traits result from multiplicative biological processes and exhibit wide variation across scales. Importantly, the same absolute change (e.g. a difference of 10 μm in vessel diameter or 2 cm in segment length) has much more significant functional and biomechanical consequences at smaller scales than at larger ones. For instance, a 10 μm increase in vessel diameter from 20 to 30 μm represents a 50% change, substantially altering hydraulic conductivity, and construction costs. By contrast, the same absolute change, from 300 to 310 μm, represents only a 3% increase, with far smaller relative effects on function. Log transformation makes these proportional differences comparable and linearizes the multiplicative relationships among variables. This facilitates interpretation of scaling patterns such as those predicted by da Vinci's rule, or Murray's law, where proportional changes in structure have nonlinear impacts on function. All statistical analyses were performed in R v.4.4.1 (R Core Team, 2024).

First, to assess the relationship between the number of functional vessels at the base and the tip of stem segments, we used standardized major axis (SMA) regression. In our system, vessel number in a stem segment reflects a response to the total leaf area supplied, and conduits at the tip and base are formed concurrently by the vascular cambium. Therefore, the number of vessels at the base does not cause the number at the tip, nor vice versa. We chose SMA rather than ordinary least squares (OLS) regression because there is no clear causal direction between these variables. OLS is appropriate when one variable clearly depends on another, whereas SMA is better suited for situations where causality is mutual (Smith, 2009). We fitted the model using the *sma()* function from the smatr package, v.3.4‐8 (Warton *et al*., 2012), with the formula vntip − vnbase, where vntip and vnbase represent the number of functional conduits at the stem tip and base, respectively. Furcation is a multiplicative hypothesis: if conduits increase in number as they narrow, tip counts should increase faster than base counts, yielding a slope > 1. The situation of no furcation predicts a slope of 1, indicating the same number of functional conduits at tip and base. A slope < 1 indicates a relative decrease toward the tip. The *y*‐intercept is not central to this question because a nonzero intercept would imply a constant additive offset that does not increase with basal conduit number or segment length, which is not a signature of furcation.

Second, if xylem conduits do significantly furcate from stem base to stem tip, then longer branches are expected to have undergone more generations of vessel proliferation, that is, more opportunities to add functional conduits. Therefore, branch length should have a positive and significant effect on the number of functional conduits at the tip. To test whether stem segment length helps predict the number of conduits at the tip, in addition to the number at the base, we used multiple linear regression. In this scenario, the *P*‐value associated with branch length in the multiple regression output should be < 0.05. Conversely, if the number of functional conduits is conserved along the branch regardless of its length, then segment length should have no significant effect on the number of conduits at the tip, resulting in a nonsignificant *P*‐value.

Theory and previous data predict that conduit diameter increases with distance from the twig tip with a slope of *c*. 0.2 (Olson *et al*., 2014, 2018, 2021; Williams *et al*., 2019). To test this prediction, we fitted a linear regression of mean vessel diameter vs distance from the twig tip, using two measurements per branch, one just below the leaves and one at the base of the branch segment. A slope close to 0.2 indicates that widening is involved in maintaining hydraulic supply without added conduit proliferation, consistent with optimality arguments about efficiency and carbon cost (Koçillari *et al*., 2021; Olson *et al*., 2021; Anfodillo & Olson, 2024). We then compared our results with the tip‐to‐base widening dataset of Koçillari *et al*. (2021) to assess whether our data overlap with previously detected patterns.

#### Phylogenetically informed SMA regression

To account for the possibility that closely related species might resemble one another more than distantly related ones in their residual values, we repeated our central analysis using a phylogenetically informed approach. We first assembled a species‐level phylogeny including all 112 species examined (Fig. 2), using v.phylomaker2 (Jin & Qian, 2022). The tree topology was based on the Angiosperm Phylogeny Group classification (The APG IV, 2016) and on the Open Tree of Life framework (Hinchliff *et al*., 2015). Species names were verified and standardized according to Plants of the World Online (POWO, 2025).

Phylogenetic dating was carried out with a penalized likelihood model implemented in the *chronos()* function of the ape package (Paradis & Schliep, 2019). *Adiantum capillus‐veneris* was used as an outgroup (ShaoGang & JinZhuang, 2013; Nitta *et al*., 2022). Missing branch lengths were estimated following Grafen's (1989) method. Temporal calibrations relied on fossil and molecular ages reported in recent literature (Stevens, 2017; Ramírez‐Barahona *et al*., 2020; Joyce *et al*., 2023; Osozawa, 2025) for 9 hierarchically nested clades: Spermatophyta, Angiospermae, Magnoliides, Monocotyledoneae, Pentapetalae, Rosids, Fabids, Asterids, Malvids. Each calibration was assigned to the most recent common ancestor of the corresponding taxa, ensuring consistency between minimum and maximum ages. Dating analyses used a correlated rates model with an empirically adjusted smoothing parameter (*λ*), yielding an ultrametric tree with branch lengths expressed in millions of years (Fig. 2).

The resulting ultrametric tree was used to phylogenetically inform our *N*_tip_–*N*_base_ regression. We fitted a phylogenetic reduced major axis (RMA) regression using the function *phyl.RMA()* in the phytools package (Revell, 2024), which estimates slopes under a Brownian motion model of trait evolution while simultaneously estimating Pagel's *λ*. This parameter scales from 0 (no phylogenetic signal, species independent) to 1 (covariance fully explained by shared ancestry).

### Comparison of empirical data with furcation models

To compare our empirical results with those that would be expected under different furcation scenarios, we used our empirical data on basal conduit number and segment length to calculate the tip conduit numbers that would be expected given different furcation scenarios. Models that invoke furcation assume that conduit proliferation happens at conduit branching events, where the relationship between the number of conduits at the base (*N*_base_) and the tip (*N*_tip_) can be written as:

(Eqn 1) $$N_{\mathrm{tip}}=N_{\text{base}}\times b^{n}$$

where *b* is the branching ratio (number of daughter conduits per parent conduit at each step), and *n* is the number of branching events that occur over the branch segment length. ‘Branch segment length’ refers to the distance between the base of the branch and the apex just below the leaves (Fig. 1).

#### Regular spacing of furcation events

To estimate *n*, assuming a regular spacing of furcation events along the segment, if the conduit internode distance per branching event is *L*_*b*_, then:

(Eqn 2) $$n=L_{\text{segment}}/L_{b}$$

where *L*_segment_ is the length of the branch segment, between the basal portion where conduit number and diameter were measured and the apical portion below the leaves. Assuming bifurcation of conduits at furcation events, estimated *N*_tip_ is

(Eqn 3) $$N_{\mathrm{tip}}=N_{\text{base}}\times 2^{n}$$

#### Decreasing conduit internode lengths base to tip

In many models of plant form and function, internode length (distance between furcation events) decreases from base to tip. The West *et al*. (1999) model posits this pattern at the branch level, and Comstock & Sperry (2000) showed that the optimal distribution of conduit tier lengths requires shorter segments distally when hydraulic efficiency is optimized subject to construction costs. Such architectures are inspired by observations from leaf vasculature and animal circulatory systems. In leaves and animal circulatory systems, the ‘capillaries’, where exchange of oxygen, CO_2_, or water occurs, the conduits are maximally narrow and highly branched. More proximally, conduits are wider and branch less for the same unit length. Whether such branching architectures are plausible for the stem has not been ascertained, but if so, vessels closer to the stem base are expected to be longer than those closer to the tip (Zimmermann & Jeje, 1981; Comstock & Sperry, 2000; Jacobsen *et al*., 2019), coinciding with empirical data; for example, tracheids near the base are longer than those at the tip, and scale with diameter (Pittermann *et al*., 2006; Sperry *et al*., 2006; Lazzarin *et al*., 2016). If conduit internode length decreases from base to tip, then the number of furcation events is no longer just *n = L*_segment_/*L*_*b*_ because this formula assumes constant internode lengths between furcation points. Instead, it is necessary to incorporate a length reduction factor that describes how much shorter daughter internodes are compared to mother conduit internodes (as in West *et al*., 1999). If conduit internode length scales by a constant factor *λ* at each furcation event, then:

(Eqn 4) $$L_{k+1}=\lambda \times L_{k}$$

where *L*_*k*_ is conduit internode length at branching level *k*, where *k* = 0 is the base internode and *k = n* the distalmost one, *L*_*k+*1_ = conduit internode length at the next branching level up from branching level *k*, and *λ* is the proportional decrease in segment length per branching event, between 0 and 1. If daughter conduit internode lengths are 70% as long as mother conduit internode lengths, then *λ* = 0.7.

The total number of branching events (*n*) needed to reach the tip is then determined by summing these successively shorter internodes. Using the formula for the sum of a finite geometric series (Mortimer, 2013), the total conductive path length from base to tip becomes:

(Eqn 5) $$L_{\text{segment}}=L_{0}\cdot \sum_{k=0}^{n}\lambda^{k}$$

where *L*_0_ is initial conduit internode length, the longest, at the base, and *L*_segment_ is total branch segment length, from the segment base to the apex below the leaves, where conduit number and diameters were measured.

Assuming *L*_*n*_ = 1 cm, then for each *L*_segment_,

(Eqn 6) $$L_{0}=\left(1-\lambda \right)\times L_{\text{segment}}+\lambda \times 1$$

We can then solve for *n*:

(Eqn 7) $$n=\log\lambda \times \left(1-\left(L_{\text{segment}}\times \left(1-\lambda \right)\right)/L_{0}\right)-1$$

we then substitute these *n*s into Eqn 3 above.

#### Conservation scenarios: da Vinci and Murray

The above furcation scenarios place no restriction on the cumulative conductive area across hierarchical levels of branching. In ‘conservation’ scenarios, a particular radius moment is conserved along the conductive path, that is, (*r*_base_/*r*_tip_)^*p*^ × *N*_base_ such that the sum over conduits of *r* ^*p*^ stays constant: the ratio *r*_base_/*r*_tip_ can be designated with *R*. For situations following area‐conservation (da Vinci's rule),

(Eqn 8) $$N_{\mathrm{tip}}=R^{2}\times N_{\text{base}}$$

representing conservation of total cross‐sectional area (Savage *et al*., 2010). For Murray's Law,

(Eqn 9) $$N_{\mathrm{tip}}=R^{3}\times N_{\text{base}}$$

minimizing pumping work subject to metabolic costs, yielding conservation of the *r* ^3^ sum (Vogel, 1996; McCulloh *et al*., 2003, 2004, 2009; McCulloh & Sperry, 2005; Gleason *et al*., 2018).

#### Implementation

Using our empirical *N*_base_ values, and in the case of da Vinci and Murray, empirical *r*_base_ and *r*_tip_ values, we implemented all three of these situations to provide baselines for comparison with our empirical results. For scenarios involving fixed internode lengths, we varied internode distances from 3 to 30 cm, in 3 cm intervals; short internodes should lead to higher *N*_tip_, and lower numbers would lead to lower *N*_tip_. For scenarios of varying internode length we assumed *λ* = 0.7. For varying internode, da Vinci, and Murray scenarios, we calculated one *N*_tip_ value per branch. We then plotted these estimated values to compare with our observed values. If conduits in stems do not furcate, then the estimates should fall above the empirical data.

## Results

### Statistical analyses

#### Standard linear models: conduit number and furcation

**Fig. 3:**
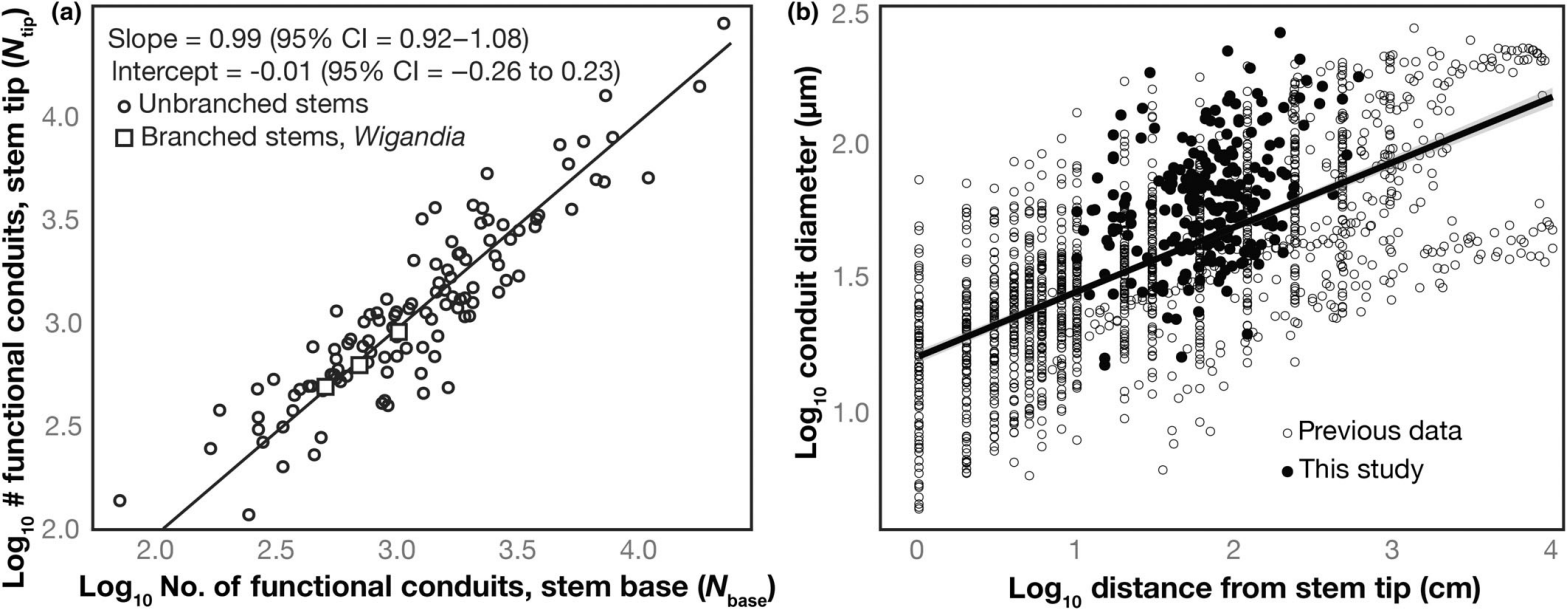
Constant numbers of conduits along stems, coincidence with tip‐to‐base widening. (a) Relationship between the number of functional xylem conduits at the stem base (*N*_base_) and at the stem tip (*N*_tip_) in log–log space. The standardized major axis (SMA) regression (solid line) yielded a slope of 0.99 (95% CI = 0.92–1.08) and an intercept of −0.01 (95% CI = −0.26 to 0.23), indicating an isometric relationship and no evidence of conduit furcation along the stem. (b) Relationship between conduit diameter and distance from the stem tip, combining data from this study (filled circles) with previously published data (open circles; Koçillari *et al*., 2021). The slope of 0.22 (95% CI = 0.15–0.31) corresponds closely to the theoretical value of 0.2 expected under hydraulically optimal conduit widening, along with previous findings of empirical slopes of *c*. 0.2, suggesting that widening rather than conduit proliferation maintains water transport along the axis. The branched stems of *Wigandia* (squares) are indistinguishable in their *N*_tip_ *–N*_base_ relations, showing that external branching does not affect conduit furcation.

The relationship between the number of functional conduits at the base and at the tip of each stem segment was strongly linear in log–log space (Fig. 3a, Table 1). The standardized major axis (SMA) regression yielded a slope of 0.99 (95% CI = 0.92–1.08) and an intercept of −0.01 (95% CI = −0.26 to 0.23; *R* ^2^ = 0.82; *P* < 2.2 × 10^−16^). The slope did not differ significantly from unity, and the intercept did not differ from zero, indicating an isometric relationship between conduit number at the base and tip. Thus, vessel number was constant along the stem, with no evidence of furcation. The *Wigandia* data from branched stems were indistinguishable from the data from branched stems, consistent with furcation being a scaling relationship between conduit number and diameter irrespective of external branching (Fig. 3a).

**Table 1:** Summary of regression models of conduit abundance and diameter.

Response variable	Predictors	Slope (95% CI)	Intercept (95% CI)	*R* ^2^	*P*
log_10_ *N*_tip_	log_10_ *N*_base_	0.99 (0.92–1.08)	−0.01 (−0.26 to 0.23)	0.82	< 2.2 × 10^−16^
log_10_ *N*_tip_	log_10_ *N*_base_ + segment length	0.90 (0.83–0.99)	0.37 (−0.25 to 0.66)	0.82	Base < 2.2 × 10^−16^; length = 0.74
log_10_ VD	log_10_ *DistTip*	0.22 (0.15–0.31)	0.00 (≈ 2 × 10^−16^)	0.12	4.3 × 10^−8^

Abbreviations: CI = 95% confidence interval; *DistTip* = distance from the stem tip (apical meristem); *N*_base_ = number of conductively active vessels at the segment tip; *N*_tip_ = number of conductively active vessels at the segment tip; VD = mean vessel diameter (μm), measured at the segment tip and base.

#### Standard linear models: conduit number, segment length and the absence of cumulative furcation

When stem segment length was included as an additional predictor of conduit number at the tip, the multiple regression model did not improve (Table 1). The effect of segment length was non‐significant (*P* = 0.74), and the confidence interval for segment length (−0.14 to 0.20) included zero, confirming that longer segments did not contain proportionally more conduits at the tip. This result reinforces the conclusion that conduit number remains effectively constant along the axis and does not accumulate with increasing branch length.

#### Standard linear models: conduit widening along the stem

As found in previous studies, conduit diameter increased significantly with distance from the branch tip (Table 1). The slope of this relationship was 0.22 (95% CI = 0.15–0.31), closely matching the theoretical value of 0.2 predicted in optimality models that balance selection favoring maintenance of conductance as path length increases with conduit construction cost and, potentially, embolism vulnerability (Olson *et al*., 2023). The 0.2 slope is also consistent with empirical values from other studies. The present data overlap entirely with previously published tip‐to‐base conduit widening profiles (Fig. 3b), supporting the interpretation that widening largely compensates for hydraulic path length without involving conduit multiplication.

#### Phylogenetically informed regressions

As expected given the nonphylogenetic results, there was no evidence of phylogenetic signal in conduit number. The phylogenetic RMA yielded an estimated *λ* of 6.6 × 10^−5^, effectively zero. *λ* ranges from 0, indicating no phylogenetic structure in the residuals (i.e. species behave as independent data points), to 1, indicating that trait covariances follow a Brownian motion model of evolution along the phylogeny. A value so close to zero therefore shows that related species do not share similar residuals beyond what is expected by chance. The phylogenetic slope (0.99) and intercept (0.004) were indistinguishable from those obtained in the standard SMA regression (slope = 0.99 (95% CI = 0.92–1.08), intercept = −0.01 (95% CI = −0.26 to 0.23); Table 1). This near‐perfect 1 : 1 relationship between the number of conduits at the base and at the tip of each stem segment implies that conduit number remains constant along the axis, leaving no scope for correlated residuals among closely related species. Accordingly, there is no basis to expect phylogenetic signal in these data, and our results confirm its absence. The lack of phylogenetic structure in conduit number parallels previous findings for conduit widening along branches (Olson & Rosell, 2013; Olson *et al*., 2014, 2018), a trait closely linked to furcation. In both cases, the strong selective pressures maintaining constant hydraulic architecture across lineages appear to override any tendency for related species to resemble one another.

### Comparison of empirical data with furcation models

**Fig. 4:**
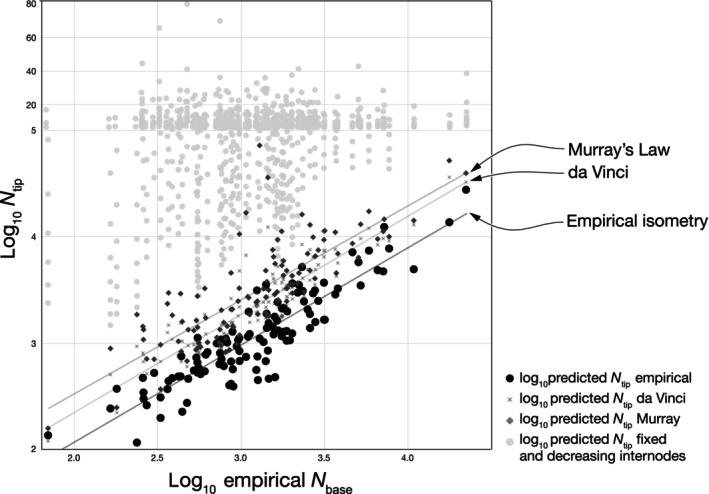
Classical furcation rules overpredict conduit numbers at the tip. Empirical data (black) show an isometric relationship between conduit number at the base (*N*_base_) and tip (*N*_tip_) of unbranched stems, indicating that plants maintain a constant number of active conduits per unit leaf area along the stem. Lines give predictions based on measured *r*_base_ and *r*_tip_ under da Vinci's rule (conservation of summed cross‐sectional area) and Murray's law (conservation of conductive power) with symmetric bifurcation; both lie above the data. Light gray points show predictions using fixed internode lengths between 3 and 30 cm over the observed segment length *L* and span even larger values. All predictions use the measured *N*_base_ and *L* for each branch measured. The *y*‐axis is log_10_; for clarity, the lower portion of the *y*‐axis has been expanded to reveal the slope of the empirical relationship; even under this scale, all theoretical predictions remain well above the observed data, emphasizing how unrealistic classical furcation or Murray‐type branching assumptions are for real stems.

Empirical tip counts scaled isometrically with basal counts across unbranched stems. All furcation baselines overpredicted *N*_tip_ (Fig. 4). Fixed internode spacing from 3 to 30 cm produced the largest overestimates, and the bias increased as spacing shortened. A geometric contraction of internode length toward the tip with *λ* = 0.7 also exceeded observations. Conservation rules using measured *r*_base_ and *r*_tip_ were closer but still high: predictions from Murray's law lay above those from da Vinci's rule, and both remained above the empirical line across the full range of *N*_base_. Together these results indicate little or no conduit multiplication along the measured stem segments and argue against furcation architectures in stems.

## Discussion

Our results suggest that the conductive system of plants is not furcated within the stem, but instead conduit number remains constant per unit leaf area tip‐to‐base. This finding supports what we have called a ‘Widened Pipe Model’, essentially a refinement of Shinozaki's classic Pipe Model (Shinozaki *et al*., 1964a, 1964b; Lehnebach *et al*., 2018; Rosell & Olson, 2019; Koçillari *et al*., 2021; Anfodillo & Olson, 2024), in which xylem conductance per unit leaf area is maintained not through the addition of new conduits distally but instead through increases in conduit diameter tip‐to‐base along the transport pathway, with a constant number of conduits per unit leaf area tip‐to‐base. The observed slope of conduit widening (0.22) closely matches theoretical expectations for hydraulically optimal scaling and aligns with previously published profiles across diverse taxa (Anfodillo *et al*., 2006; Coomes *et al*., 2007; Olson & Rosell, 2013; Olson *et al*., 2014, 2020; Petit *et al*., 2014, 2022, 2023; Lazzarin *et al*., 2016; Williams *et al*., 2019; Koçillari *et al*., 2021; Bok *et al*., 2022; Fajardo *et al*., 2022; Chambers‐Ostler *et al*., 2023; Rita *et al*., 2024; Zambonini *et al*., 2024; Khoma & McAdam, 2025; Simovic & Michaletz, 2025), indicating that widening is a general mechanism sustaining conductance across terrestrial plants, without invoking conduit multiplication. The Widened Pipe Model thus reconciles the empirical constancy of conduit number with selection favoring constant water transport per unit crown area, without the need to invoke furcation assumptions.

As in the Widened Pipe Model, our results are consistent with the notion that furcation occurs only in the leaves, not within the transport tissues of the stem (Mencuccini *et al*., 2007; Price *et al*., 2013; Gleason *et al*., 2018; Lechthaler *et al*., 2019; Rosell & Olson, 2019). The distal portion of a plant's conductive system, which can be referred to as the diffusive domain, is confined to the finest leaf veins, where conduits divide and terminate near the evaporation sites in the mesophyll (Lechthaler *et al*., 2019; Rosell & Olson, 2019; Sopp & Valbuena, 2023). Proximal to this, in the transport domain encompassing the petiole and stem, conduit number remains essentially constant while diameters widen toward the base, forming a nonfurcated pathway that conducts water over long distances. The distinction between these domains reflects a shift from a branching network in the leaves to a continuous transport system in the stem.

The absence of furcation in stems implies that natural selection acts against hydraulic architectures in which conduit number increases toward the stem tip. For stems with the same number of conduits at the base, a furcated system would require a larger total conductive wall area, leading to a higher carbon cost per unit leaf area. Several authors have noted that such systems would be structurally and energetically disadvantageous: McCulloh & Sperry (2005) emphasized that increased conduit proliferation elevates construction costs, and Denny (2012) even suggested that excessive distal widening or branching would produce ‘top‐heavy’ stems prone to mechanical failure given excessive distal weight. The consistent absence of furcation therefore indicates that widening, including any permeability increases associated with ultra‐widening patterns (Anfodillo & Olson, 2024), is sufficient to maintain conductance to the leaves without requiring extensive conduit proliferation along stems.

Beyond its carbon cost, selection also seems unlikely to favor strong basal coalescence into a few ‘aorta‐like’ conduits, because such coalescence would impede sectoriality (Larson *et al*., 1994; Orians *et al*., 2005; Zanne *et al*., 2006; Nadezhdina, 2010; David *et al*., 2012; McElrone *et al*., 2021). In this context, ‘sectoriality’ refers to different areas of the crown transpiring at markedly different rates. The well‐documented differential response of trees to spatial and diurnal heterogeneity in light, in which one sector of the crown demands peak flow in the morning while another peaks in the afternoon, are an example. In such canopies, individual leaves experience rapid, local changes in radiation and wind, so water demand varies leaf by leaf; forcing many leaves to share an upstream conduit couples hydraulics that must remain independent. By contrast, a layout with relatively independent parallel conduits allows each leaf to be supplied in proportion to its instantaneous demand regardless of neighbors' conditions. In short, both selection favoring redundancy and favoring transpirational tracking of fine‐scale, time‐varying demand argue against highly furcating topologies in the stem, and instead favor architectures that maintain axial isolation of flow paths to terminal organs.

Given the empirical absence of furcation, and even compelling theory showing that conduit proliferation is not only unnecessary for conductance to remain constant to the leaves but would likely incur carbon penalties, we conclude by turning to a likely reason that conduits in stems have so often been assumed to furcate.

### Da Vinci and furcation

We suspect that one reason that including furcation plant structure–function models has proven so appealing is the concomitant acceptance of da Vinci's ‘rule’ of area‐preserving branching as applied to stem cross‐sectional area. Da Vinci's rule consists of two postulates: first, that the summed cross‐sectional area of daughter branches equals that of their parent branch, and second, that stems do not taper between successive branching points (Sone *et al*., 2009; Eloy, 2011; Chen *et al*., 2012; Bentley *et al*., 2013; Minamino & Tateno, 2014; Grigoriev *et al*., 2022).

Reconciling this ‘rule’ with what one observes under the microscope is problematic. In a section of wood, when conduits are narrow, conduit density is high, that is, there are many per unit xylem cross sectional area, for example, conduits·mm^−2^. When conduits are wide, density is low and there are few per unit area. Because of tip‐to‐base conduit widening in the stems of woody plants, conduits are much narrower at the treetop twigs than at the base (Anfodillo & Olson, 2021), so conduits·mm^−2^ is vastly higher at the twig tip than at the trunk base. To calculate the total number of conduits, one must multiply total sapwood cross sectional area by conduits·mm^−2^. Given that da Vinci's rule postulates that sapwood area is the same at the base and across the summed twig tips, biologists are faced with a huge problem to account for: multiplying apical conduit density by basal sapwood implies that there must be much higher total conduit numbers at the tip than at the base. So, conduit furcation is introduced as a patch.

Given our results here and that tip‐to‐base conduit widening plus any ultra‐widening permeability increases (Anfodillo & Olson, 2024) seem perfectly capable of maintaining leaf‐specific conductance with height growth at much lower carbon cost, it seems more likely that trees depart from da Vinci and that there is less total sapwood area at the tip than at the base. Like furcation, da Vinci's rule is widely invoked in models of tree structure and function (Yamamoto, 1994; Hatsch, 1997; Horn, 2000; Oppelt *et al*., 2001; McCulloh *et al*., 2003; Sone *et al*., 2005, 2009; Sperry *et al*., 2008, 2012; Savage *et al*., 2010; Eloy, 2011; Chen *et al*., 2012; Bentley *et al*., 2013; Price *et al*., 2013, 2022; Minamino & Tateno, 2014; Newberry *et al*., 2015; Couvreur *et al*., 2018; Lehnebach *et al*., 2018; Lechthaler *et al*., 2019; Sotolongo‐Costa *et al*., 2020; Gao & Newberry, 2025), and like furcation in the stem it lacks extensive empirical support (Nikinmaa, 1992; Yamamoto, 1994; Horn, 2000; Sone *et al*., 2005; Bettiati *et al*., 2012; Bentley *et al*., 2013; Minamino & Tateno, 2014; Price *et al*., 2022). Our data thus suggest that it is likely that total sapwood area at the tips of plants is less than at the base, and show that, rather than a furcated network, plant hydraulic architecture in the stem is better represented by a Widened Pipe Model.

## Competing interests

None declared.

## Author contributions

GA‐S, TA and MEO designed the research; GA‐S, AIP‐M and MEO carried out the research; GA‐S collected the data; GA‐S, MMA‐H and MEO analyzed the data; all authors participated in writing the manuscript.

## Disclaimer

The New Phytologist Foundation remains neutral with regard to jurisdictional claims in maps and in any institutional affiliations.

## Supporting information


**Dataset S1** Dataset containing the counts of functional xylem conduits and conduit diameters for both basal and apical branch segments as well as total branch length for each of the 112 species.

**Table S1** List of species from which branches were collected to analyze the number of functional xylem conduits at the base and apex, just below the point where leaves or secondary branches emerged.
Please note: Wiley is not responsible for the content or functionality of any Supporting Information supplied by the authors. Any queries (other than missing material) should be directed to the *New Phytologist* Central Office.

## Data Availability

The data supporting the findings of this study, including a list of studied species (Table S1), the dataset containing counts of functional xylem conduits per species (Dataset S1), measurements of conduit diameters and branch length, as well as all photographs used to obtain quantitative variables, are openly available in Figshare, at https://doi.org/10.6084/m9.figshare.30569648. The photographs provide a photographic record used to count functional xylem conduits in the 112 vascular plant species included in this study. For each species, the image corresponds to a transverse section of the basal and apical segment of a branch, taken just below the point of leaf or secondary branch emergence.
