# Supplementary material for: Da Vinci's mischief: xylem conduits in the stems of woody plants do not furcate
Source: New Phytol. 2026 Mar 24;250(5):3001–13. doi: 10.1111/nph.71097 (PMC13150319; doi:10.1111/nph.71097)
Supplement: Supplementary file 2 — Table S1 List of species from which branches were collected to analyze the number of functional xylem conduits at the base and apex, just below the point where leaves or secondary branches emerged. Please note: Wiley is not responsible for the content or functionality of any Supporting Information supplied by the authors. Any queries (other than missing material) should be directed to the New Phytologist Central Office. [file NPH-250-3001-s002.pdf]

New Phytologist Supporting Information

Article title: **Da Vinci's mischief: xylem conduits in the stems of woody plants do not furcate**

Authors: Gilberto Alemán-Sancheschúlz, Tommaso Anfodillo, Ana Isabel Pérez-Maussán, María Magdalena Ayala-Hernández, Mark E. Olson.

Article acceptance date: 18 February 2026.

SUPPLEMENTARY MATERIAL

Table S1. List of species from which branches were collected to analyze the number of functional xylem conduits at the base and apex, just below the point where leaves or secondary branches emerged. A total of 112 species of vascular plants were collected, representing 57 families and 31 orders. Branches from 41 species were collected at the Los Tuxtlas Field Station (Veracruz, México), 55 species at the Chamela Field Station (Jalisco, México), and 16 species at the Francisco Javier Clavijero Botanical Garden of the Instituto de Ecología A.C. (INECOL; Veracruz, México).

**Order**	**Family**	**Species**	**Locality**
Malpighiales	Euphorbiaceae	*Acalypha diversifolia* Jacq.	Los Tuxtlas
Malpighiales	Euphorbiaceae	*Acalypha langiana* Müll.Arg.	Chamela
Malpighiales	Euphorbiaceae	*Acalypha skutchii* I.M.Johnst.	Los Tuxtlas
Sapindales	Sapindaceae	*Acer skutchii* Rehder	INECOL
Malpighiales	Euphorbiaceae	*Adelia barbinervis* Schltdl. & Cham.	Los Tuxtlas
Santalales	Opiliaceae	*Agonandra racemosa* (DC.) Standl.	Chamela
Sapindales	Sapindaceae	*Allophylus camptostachys* Radlk.	Los Tuxtlas
Sapindales	Anacardiaceae	*Amphipterygium adstringens* (Schltdl.) Schiede ex Standl.	Chamela
Fabales	Fabaceae	*Apoplanesia paniculata* C.Presl	Chamela
Ericales	Primulaceae	*Ardisia compressa* Kunth	Los Tuxtlas
Piperales	Aristolochiaceae	*Aristolochia taliscana* Hook. & Arn.	Chamela
Sapindales	Anacardiaceae	*Astronium graveolens* Jacq.	Chamela
Austrobaileyales	Austrobaileyaceae	*Austrobaileya scandens* C.T.White	INECOL
Lamiales	Acanthaceae	*Avicennia germinans* (L.) L.	INECOL
Rosales	Moraceae	*Brosimum alicastrum* Sw.	Los Tuxtlas
Sapindales	Burseraceae	*Bursera instabilis* McVaugh & Rzed.	Chamela
Sapindales	Burseraceae	*Bursera palaciosii* Rzed. & Calderón	Chamela
Sapindales	Burseraceae	*Bursera simaruba* (L.) Sarg.	Chamela
Fabales	Fabaceae	*Caesalpinia pulcherrima* (L.)Sw.	Chamela
Metteniusales	Metteniusaceae	*Calatola costaricensis* Standl.	Los Tuxtlas
Brassicales	Caricaceae	*Carica papaya* L.	INECOL
Gentianales	Apocynaceae	*Cascabela ovata* (Cav.) Lippold	Chamela
Rosales	Urticaceae	*Cecropia obtusifolia* Bertol.	Chamela
Malvales	Malvaceae	*Ceiba speciosa* (A.St.-Hil., A.Juss. & Cambess.) Ravenna	Chamela
Fabales	Fabaceae	*Cenostigma eriostachys* (Benth.) Gagnon & G.P.Lewis	Chamela
Arecales	Arecaceae	*Chamaedorea elegans* Willd.	INECOL
Malvales	Bixaceae	*Cochlospermum vitifolium* (Willd.) Spreng.	Chamela
Gentianales	Rubiaceae	*Coffea arabica* L.	INECOL
Boraginales	Boraginaceae	*Cordia elaeagnoides* A.DC.	Chamela

Lamiales	Bignoniaceae	*Crescentia alata* Kunth	Chamela
Asterales	Asteraceae	*Critonia daleoides* DC.	Los Tuxtlas
Malpighiales	Euphorbiaceae	*Croton suberosus* Kunth	Chamela
Malpighiales	Euphorbiaceae	*Croton xalapensis* L.	INECOL
Laurales	Lauraceae	*Damburneya ambigens* (S.F.Blake) Trofimov	Los Tuxtlas
Laurales	Lauraceae	*Damburneya salicifolia* (Kunth) Trofimov & Rohwer	Los Tuxtlas
Malvales	Thymelaeaceae	*Daphnopsis megacarpa* Nevling & Barringer	Los Tuxtlas
Apiales	Araliaceae	*Dendropanax arboreus* (L.) Decne. & Planch.	Los Tuxtlas
Asparagales	Asparagaceae	*Dracaena fragrans* (L.) Ker Gawl.	INECOL
Fabales	Fabaceae	*Entada polystachya* (L.) DC.	Chamela
Rosales	Rosaceae	*Eriobotrya japonica* (Thunb.) Lindl.	INECOL
Myrtales	Myrtaceae	*Eugenia acapulcensis* Steud.	Los Tuxtlas
Myrtales	Myrtaceae	*Eugenia oerstediana* O.Berg	Los Tuxtlas
Brassicales	Resedaceae	*Forchhammeria pallida* Liebm.	Chamela
Ginkgoales	Ginkgoaceae	*Ginkgo biloba* L.	INECOL
Rosales	Rhamnaceae	*Gouania lupuiloides* (L.) Urb.	Chamela
Caryophyllales	Nyctaginaceae	*Guapira petenensis* (Lundell) Lundell	Chamela
Sapindales	Meliaceae	*Guarea glabra* Vahl	Los Tuxtlas
Sapindales	Meliaceae	*Guarea guidonia* (L.) Sleumer	Los Tuxtlas
Malvales	Malvaceae	*Guazuma ulmifolia* Lam.	Chamela
Laurales	Hernandiaceae	*Gyrocarpus jatrophifolius* Domin **falta TIP**	Chamela
Chloranthales	Chloranthaceae	*Hedyosmum mexicanum* C.Cordem.	Chamela
Malvales	Malvaceae	*Heliocarpus pallidus* Rose	Chamela
Ranunculales	Menispermaceae	*Hyperbaena ilicifolia* Standl. **falta TIP**	Chamela
Fabales	Fabaceae	*Inga acrocephala* Steud.	Los Tuxtlas
Solanales	Convolvulaceae	*Ipomoea wolcottiana* Rose	Chamela
Caryophyllales	Amaranthaceae	*Iresine arbuscula* Uline & W.L.Bray	Los Tuxtlas
Malpighiales	Violaceae	*Ixchelia mexicana* (Ging. ex DC.) H.E.Ballard & Wahlert	Chamela
Malpighiales	Euphorbiaceae	*Jatropha chamelensis* Pérez-Jim.	Chamela
Malpighiales	Euphorbiaceae	*Jatropha standleyi* Steyerm.	Chamela
Malpighiales	Euphorbiaceae	*Jatropha villosa* Wight	Chamela
Lamiales	Verbenaceae	*Lippia origanoides* Kunth	Chamela
Fabales	Fabaceae	*Lonchocarpus eriocarinalis* Kunth	Chamela
Magnoliales	Magnoliaceae	*Magnolia dealbata* Zucc.	INECOL
Malpighiales	Euphorbiaceae	*Manihot chlorosticta* Standl. & Goldman	Chamela
Malpighiales	Euphorbiaceae	*Manihot obovata* J.Jiménez Ram.	Chamela
Gentianales	Apocynaceae	*Matelea lanata* (Zucc.) Woodson	Chamela
Sapindales	Meliaceae	*Melia azedarach* L.	INECOL
Brassicales	Moringaceae	*Moringa drouhardii* Jum.	Chamela
Brassicales	Moringaceae	*Moringa oleifera* Lam.	Chamela
Brassicales	Moringaceae	*Moringa stenopetala* (Baker f.) Cufod.	Chamela
Brassicales	Capparaceae	*Morisonia indica* (L.) ined.	Chamela
Brassicales	Capparaceae	*Morisonia tuxtlensis* (Cornejo & Iltis) Christenh. & Byng	Los Tuxtlas
Myrtales	Myrtaceae	*Myrcianthes fragrans* (Sw.) McVaugh	Los Tuxtlas

Rosales	Urticaceae	*Myriocarpa longipes* Liebm.	Los Tuxtlas
Metteniusales	Metteniusaceae	*Oecopetalum mexicanum* Greenm. & C.H.Thomps.	INECOL
Malpighiales	Violaceae	*Orthion oblanceolatum* Lundell	Los Tuxtlas
Malpighiales	Ochnaceae	*Ouratea theophrasta* (Planch.) Baill.	Los Tuxtlas
Malvales	Malvaceae	*Pachira aquatica* Aubl.	Chamela
Gentianales	Rubiaceae	*Palicourea tetragona* (Donn.Sm.) C.M.Taylor	Los Tuxtlas
Sapindales	Sapindaceae	*Paullinia clavigera* Schltdl.	Chamela
Ericales	Sapotaceae	*Peteniodendron durlandii* (Standl.) Lundell	Los Tuxtlas
Piperales	Piperaceae	*Piper auritum* Kunth	Los Tuxtlas
Piperales	Piperaceae	*Piper hispidum* Sw.	Los Tuxtlas
Malpighiales	Picrodendraceae	*Piranhea mexicana* (Standl.) Radcl.-Sm.	Chamela
Gentianales	Apocynaceae	*Plumeria rubra* L.	Chamela
Pinales	Podocarpaceae	*Podocarpus matudae* Lundell	INECOL
Ericales	Sapotaceae	*Pouteria rhynchocarpa* T.D.Penn.	Los Tuxtlas
Ericales	Sapotaceae	*Pouteria sapota* (Jacq.) H.E.Moore & Stearn	INECOL
Rosales	Urticaceae	*Pouzolzia occidentalis* Gaudich. **falta TIP**	Chamela
Myrtales	Myrtaceae	*Psidium oligospermum* Mart. ex DC.	Chamela
Malvales	Malvaceae	*Quararibea yunckeri* Standl.	Los Tuxtlas
Gentianales	Rubiaceae	*Randia malacocarpa* Standl.	Chamela
Malpighiales	Violaceae	*Rinorea guatemalensis* (S.Watson) Bartlett	Los Tuxtlas
Lamiales	Bignoniaceae	*Roseodendron donnell-smithii* (Rose) Miranda	Chamela
Proteales	Proteaceae	*Roupala montana* Aubl.	Los Tuxtlas
Oxalidales	Connaraceae	*Rourea glabra* Kunth	Chamela
Caryophyllales	Polygonaceae	*Ruprechtia fusca* Fernald	Chamela
Celastrales	Celastraceae	*Salacia megistophylla* Standl.	Los Tuxtlas
Laurales	Siparunaceae	*Siparuna thecaphora* (Poepp. & Endl.) A.DC.	Los Tuxtlas
Sapindales	Anacardiaceae	*Spondias purpurea* L.	Chamela
Caryophyllales	Stegnospermataceae	*Stegnosperma cubense* A.Rich.	Chamela
Gentianales	Apocynaceae	*Tabernaemontana arborea* Rose	Los Tuxtlas
Sapindales	Anacardiaceae	*Tapirira mexicana* Marchand	Los Tuxtlas
Sapindales	Sapindaceae	*Thouinia paucidentata* Radlk. ex Millsp.	Chamela
Sapindales	Sapindaceae	*Thouinidium decandrum* (Bonpl.) Radlk.	Chamela
Rosales	Moraceae	*Trophis glabrata* (Liebm.) C.C.Berg	Los Tuxtlas
Crossosomatales	Staphyleaceae	*Turpinia occidentalis* (Sw.) G.Don	Los Tuxtlas
Rosales	Urticaceae	*Urera baccifera* Gaudich.	INECOL
Rosales	Urticaceae	*Urera caracasana* (Jacq.) Gaudich. ex Griseb.	Los Tuxtlas
Rosales	Urticaceae	*Urera glabriuscula* V.W.Steinm.	Los Tuxtlas
Magnoliales	Myristicaceae	*Virola guatemalensis* (Hemsl.) Warb.	Los Tuxtlas
Myrtales	Vochysiaceae	*Vochysia guatemalensis* Donn.Sm.	Los Tuxtlas
